# The Volar Central Approach for Distal Radius Fracture: A Prospective Nerve Conduction Study of 38 Patients

**DOI:** 10.1177/15589447251366459

**Published:** 2025-09-07

**Authors:** Hugo Jakobsson, Eva Lundqvist, Sara Nordkvist, Kathe Dahlbom, Marcus Sagerfors

**Affiliations:** 1 Department of Hand and Orthopedic Surgery, Faculty of Medicine and Health, Örebro University, Sweden; 2 School of Health Sciences, Örebro University, Sweden; 3 Department of Neurophysiology, Örebro University Hospital, Sweden

**Keywords:** distal radius fracture, median nerve, nerve conduction study, volar central approach

## Abstract

**Background:**

In distal radius fracture (DRF) surgery with volar locking plates, the flexor carpi radialis approach is commonly used. However, the volar central approach (VCA), between the median nerve and the finger flexors, may improve visualization of the volar ulnar corner. A similar approach has been linked with a higher risk of iatrogenic median neuropathy. This study evaluated the risk of median neuropathy after operative treatment using the VCA.

**Methods:**

Thirty-eight patients with Arbeitsgemeinschaft für Osteosynthesefragen (AO) type C DRF were assessed prospectively with sensory nerve conduction studies preoperatively and at 6 weeks, 3 months, and 12 months postoperatively.

**Results:**

At 6 weeks, 30 of the 38 patients exhibited median neuropathy, decreasing to 12 of the 35 at 12 months. Subjective sensory deficit was reported by 12 of the 38 patients at 6 weeks and 8 of the 37 at 12 months. Patients with median neuropathy (MN) had significantly higher frequency of subjective sensory deficit of the median nerve 12 months postoperatively, but did not have significantly worse patient-reported outcome.

**Conclusions:**

Our results suggest that the VCA should be reserved for cases needing optimal exposure of the volar ulnar corner.

## Introduction

Distal radius fracture (DRF) is the most common type of fracture in adults.^1^ Many cases can be managed conservatively, but displaced fractures often require surgical treatment.^2^ Since the introduction of the volar locking plate (VLP) over 2 decades ago,^3^ this has become the dominant surgical treatment modality.^2^ The VLP has been compared with other surgical treatment methods in several studies, and is known to be a feasible option in the majority of cases.^4-6^

The flexor carpi radialis (FCR) approach is commonly used to apply the VLP, providing surgical access to the fracture between the FCR tendon and the radial artery. This approach can be extended distally over the wrist flexion crease if the fracture system demands better exposure.^7^ However, even with the extended FCR approach, adequate exposure of the volar ulnar corner can be cumbersome.^8^ Stabilizing the fragments of the volar ulnar corner, often referred to as the “critical corner,” is essential for successful fracture treatment.^8,9^ In Arbeitsgemeinschaft für Osteosynthesefragen (AO) type C3 DRFs,^10^ fracture fragments in this area has been reported to be the dominant reason for loss of reduction.^11^

An alternative approach is the volar central approach (VCA). This approach visualizes the fracture between the tendons of the finger flexors, which are retracted ulnarly, and the median nerve, the tendons of the FCR, and the flexor pollicis longus, which are retracted radially. It enables excellent visualization of the ulnar part of the radius, thus facilitating adequate reduction and fixation of the volar ulnar corner fragment.^12^ However, one retrospective study^13^ raised concerns regarding a similar approach, the volar ulnar approach (VUA), where the fracture is reached ulnar to the finger flexors.^14^ The authors noted a pronounced higher frequency of median neuropathy (MN) at 6 weeks postoperatively and a slightly higher frequency at 1 year postoperatively. The benefits of excellent visualization must therefore be weighed against the unknown risk of iatrogenic MN. To our knowledge, no prospective study using nerve conduction studies has been conducted to evaluate the VCA for VLP surgery.

The aim of this study was to study MN after an intraarticular DRF treated surgically with a volar locking plate via the VCA using nerve conduction studies preoperatively and up to 1 year postoperatively. Secondary outcomes included range of motion, grip strength, VAS pain scores, patient-reported outcome measures (PROMs), and radiographic outcome.

## Methods

Approval for this study was obtained from the Swedish Ethical Review Authority (reference number 2020-0155), written informed consent was obtained from all participants, and the tenets of the Helsinki Declaration were adhered to. The study was conducted at the Department of Hand Surgery at Örebro University Hospital in Sweden.

Participants were enrolled in this prospective cohort study between January and December 2020. The inclusion criteria were being aged 18 to 70 years and having an AO type C DRF with either ≥20° dorsal angulation, >2 mm shortening, or >1 mm incongruency in the articular surface of the radiocarpal or distal radioulnar joint. Exclusion criteria were previous DRF on the same side, open fracture, diaphyseal fracture extension, bilateral fractures, ipsilateral concomitant upper extremity fracture, surgery > 12 days after the fracture, dementia, mental illness, alcohol or drug abuse, difficulty understanding the Swedish language, diabetes, previous carpal tunnel surgery, or a diagnosed and symptomatic carpal tunnel syndrome.

Data recorded by the attending physician at inclusion were: trauma mechanism (categorized as high or low energy, with low defined as a simple fall of < 2 m), sex, subjective sensory deficit (SSD; defined as numbness or paresthesia in the median nerve sensory area), and excessive swelling. A registered biomedical scientist at the Department of Neurophysiology conducted preoperative sensory nerve conduction studies (sNCS) and Semmes-Weinstein monofilament testing (SWMT). The SWMT was performed according to the manual for assessment of hand function after nerve repair published by the Swedish national quality register for hand surgery, assessing sensibility at 3 locations: the pulp of the thumb and index finger, and the palmar aspect of the base of the index finger.^15^ Each site was scored on a scale from 0 to 5, yielding a total score ranging from 0 to 15. To standardize results, the score on the injured side was expressed as a percentage of the score on the uninjured side.

At 6 weeks, 3 months, and 12 months postoperatively, the following data were recorded: SSD, the patient’s scores on the validated Swedish translations of the Patient-Rated Wrist Evaluation (PRWE)^16^ and the Quick Disabilities of the Arm, Shoulder, and Hand (QuickDASH),^17^ visual analogue pain score (VAS) at rest and during activity, hand grip strength, and wrist range of motion (ROM). Grip strength was expressed as percentage of the uninjured side. The sNCS and SWMT were performed at the Department of Neurophysiology at 6 weeks, 3 months, and 12 months postoperatively. The primary outcome was the presence of MN on sNCS 6 weeks, 3 months, and 12 months postoperatively. Radiographs (anteroposterior and lateral views) were performed preoperatively, perioperatively, and at 2 weeks, 3 months, and 12 months postoperatively. Soong et al^18^ grade was assessed on the 12-month postoperative radiographs.

### Surgical Technique and Postoperative Regimen

**Figure 1.**
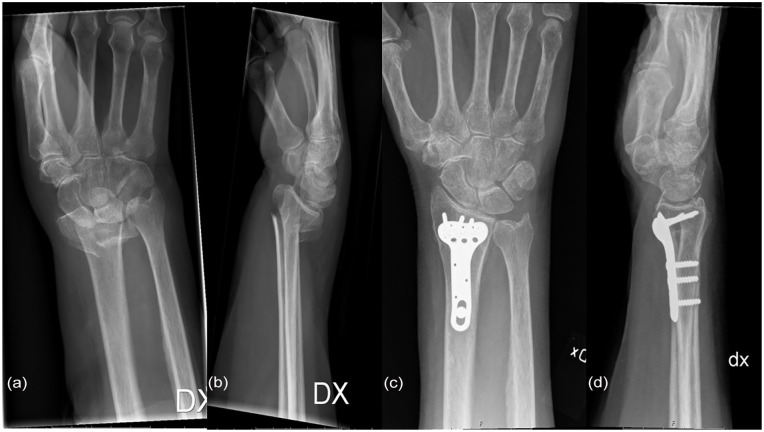
(a) Anteroposterior view of preoperative radiograph. (b) Lateral view of preoperative radiograph. (c) Anteroposterior view of 12-month postoperative radiograph. (d) Lateral view of 12-month postoperative radiograph.

**Figure 2.**
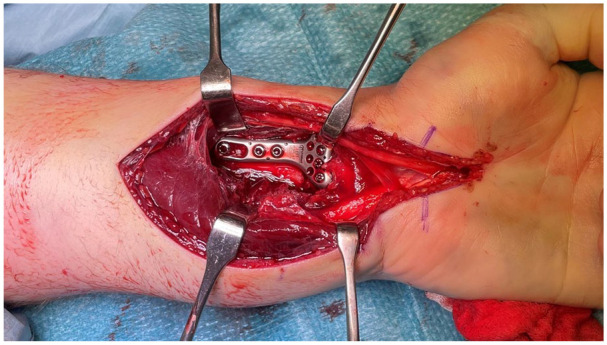
Distal radius fracture exposed, repositioned, and fixed with a volar locking plate via the volar central approach.

All procedures were performed by, or under supervision of, a board certified specialist hand surgeon (experience level 3-4 according to Tang and Giddins^19^) under general anesthesia with a brachial plexus block and a bloodless field maintained with a pneumatic tourniquet on the upper arm. At the time of the study, the VCA was the standard approach of the department for AO type C fractures. An incision was made over the carpal tunnel and distal central volar forearm, crossing the flexor crease of the wrist in a zigzag fashion. Next, the carpal ligament and antebrachial fascia were divided longitudinally. The median nerve was identified and retracted radially together with the tendons of the FCR and flexor pollicis longus, and the flexors of the fingers were retracted ulnarly. The pronator quadratus was divided and elevated, and the fracture was exposed. After reduction of the fracture, the VLP (TriMed Inc, Santa Clarita, California) was applied (Figures 1 and 2). The pronator quadratus was repaired if feasible, using absorbable sutures. After wound closure with non-absorbable sutures, a volar wrist splint was applied.

On the day after the surgery, the patient met with a hand therapist for finger mobilization exercises and edema control, and was then discharged from the hospital. After 2 weeks, the splint was removed and gentle wrist mobilization exercises were initiated under the supervision of a hand therapist. The patient was given a wrist orthosis to be used between the exercise sessions. Full loading was permitted at 3 months postoperatively.

### Sensory Nerve Conduction Studies

The sNCS to assess MN was performed by a registered biomedical scientist using an electroneurography setup (Keypoint Net, Dantec, Skovlunde, Denmark). The majority of the examinations were performed by one of the present authors (S.N.). The preoperative sNCS took place with the fractured wrist immobilized in a splint. Antidromic stimulation of the median nerve was performed at the proximal forearm, with recordings made on the third digit. Supramaximal stimulation, which is used to ensure maximal activation of all sensory nerve fibers, was not feasible in all cases preoperatively due to patient discomfort. Preoperative examinations were conducted between 0 and 12 days after the fracture, with a median of 4 days (interquartile range [IQR]: 2-8).

The sNCS at 6 weeks, 3 months, and 12 months postoperatively was performed without a splint. Orthodromic stimulation was performed by stimulating the third and fourth digits, with recordings obtained at the wrist, at fixed intervals from the stimulation point. Supramaximal stimulation was successfully achieved in all postoperative sNCS.

Median neuropathy was defined as amplitude reduction of ≥50% compared with the uninjured side; which is the routine at the Department of Neurophysiology, Örebro University Hospital.

### Statistical Analysis

Descriptive statistics were calculated as median with IQR for continuous variables and count with percentage for categorical variables.

Normality of continuous variables was assessed using the Shapiro-Wilk test (data not shown). All tested variables were found to deviate from normality, supporting the use of non-parametric tests.

To investigate whether AO classification or high/low energy trauma mechanism were related to MN on sNCS preoperatively, the Fisher-Freeman-Halton exact test was used for AO classification, while Fisher’s exact test was used for the binary trauma mechanism variable.

To determine whether patients with MN at 6 weeks, 3 months, and 12 months postoperatively experienced worse outcomes, we divided the cohort according to the presence or absence of MN at each respective follow-up time. The subgroups were then compared regarding PRWE, QuickDASH, VAS at rest and during activity, grip strength percentage, SSD, and SWMT percentage. Continuous variables were compared using the Mann-Whitney U test, while categorical variables were compared using Fisher’s exact test.

A significance level (α) of .05 was applied to all statistical tests.

## Results

**Figure 3.**
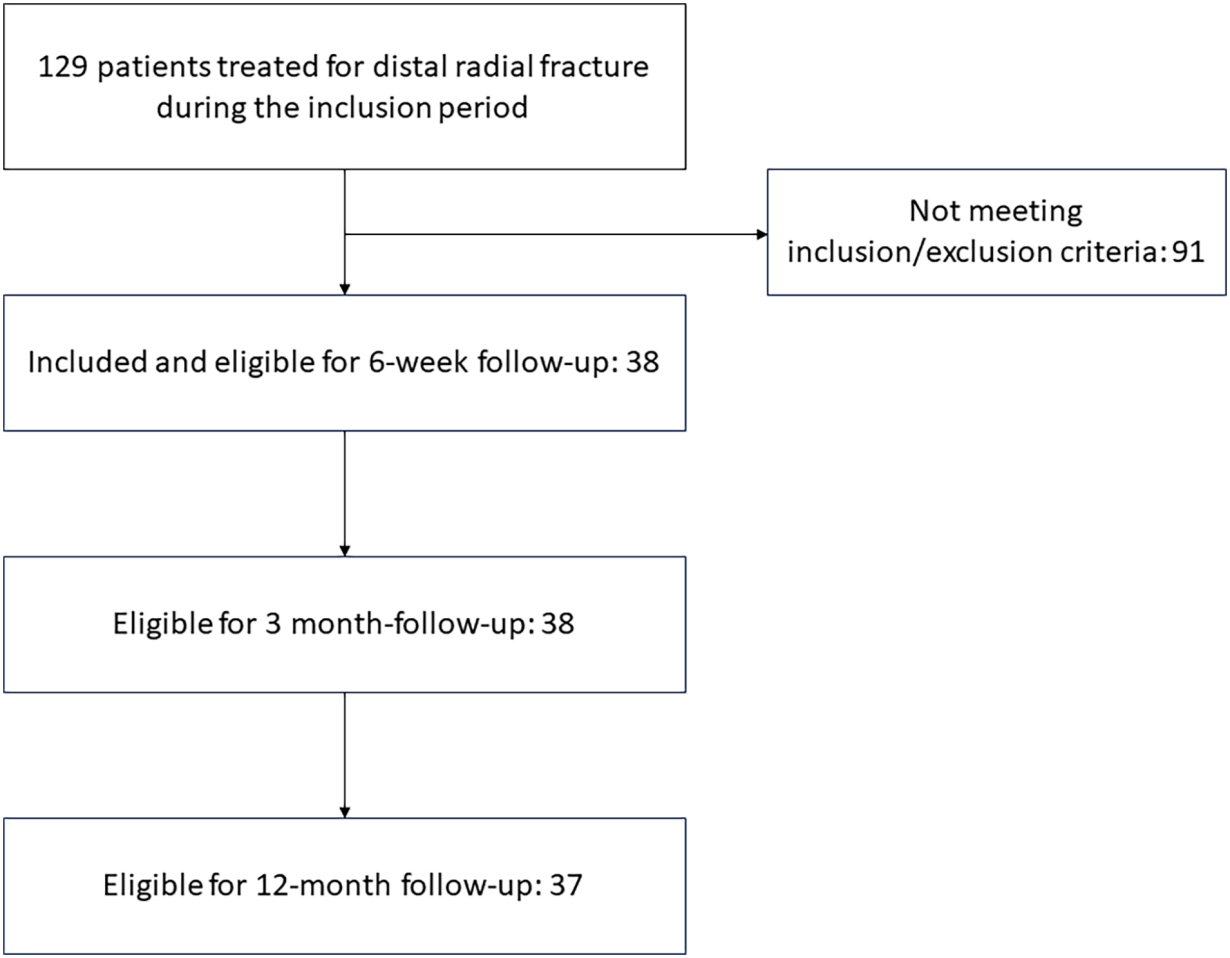
Details of patient inclusion.

A total of 38 patients were included in the study; Figure 3 shows details of patient inclusion. All patients completed the 6-week and 3-month follow-ups. One patient died during the follow-up period, leaving 37 patients eligible for the 12-months follow-up. Valid sNCS results were obtained for 35 patients preoperatively, 38 patients at the 6-week follow-up, 37 patients at the 3-month follow-up, and 35 patients at the 12-month follow-up. Details of patient inclusion are given in Figure 1, the characteristics of the cohort are presented in Table 1, and the outcome measures are presented in Tables 2 and 3.

**Table 1.** Characteristics of the Cohort.

Variable	n/median	%/interquartile range
Age (years)	62.5	55.75-70
Female sex	34	89.5
Fracture type		
AO type C1	4	10.5
AO type C2	19	50.0
AO type C3	15	39.5
High energy trauma mechanism	3	7.9
Excessive swelling at inclusion	7	18.4
Preoperative radiological parameters		
Anteroposterior ad latus displacement (mm)	0	−3 to 7.25
Dorsal angulation (°)	111.5	88.5-127.8
Ulnar height (mm)	0	0-4
Radial inclination (mm)	99	90-103.25
Postoperative radiological parameters		
Soong grade		
Grade 0	26	68.4
Grade 1	12	31.6
Grade 2	0	0
Dorsal angulation (°)	85	83-90
Ulnar height (mm)	0	−2 to 0
Radial inclination (°)	109	106.8-112.3
Operation time (minutes)	59	46.3-73

*Note.* Data are presented as n and percentage for categorical variables or median and interquartile range for continuous variables. AO = Arbeitsgemeinschaft für Ostheosynthesefragen.

**Table 2.** Outcome Measures Preoperatively and at the 6-Week, 3-Month, and 12-Month Follow-Ups.

Variable	Preoperative	6 weeks	3 months	12 months
MN on sNCS	5/35	30/38	19/37	12/35
MN on sNCS (preop. MN excluded)		26/33	15/32	9/30
SWMT percentage	100 (93-107)	100 (100-107)	100 (100-107)	100 (100-100)
Subjective sensory deficit	3/38	12/38	11/38	8/37
QuickDASH	-	35.25 (26-56)	22.7 (9.1-35)	6.8 (2.3-22)
PRWE	-	38.8 (25-63)	20.3 (13-36)	7.3 (2.8-17)
VAS at rest	-	0.7 (0-2.0)	0 (0-1.0)	0 (0-0)
VAS during activity	-	3 (0.6-5.3)	1.9 (0.7-3.1)	1 (0-3.0)
Grip strength percentage	-		56 (43-73)	84 (69-89)
Flexion		50 (40-55)	58 (50-65)	70 (60-75)
Extension		25 (20-40)	45 (35-50)	55 (45-60)
Ulnar deviation		20 (19-26)	25 (20-30)	30 (25-30)
Radial deviation		15 (10-20)	15 (15-20)	20 (18-25)
Pronation		70 (65-75)	75 (70-80)	75 (70-85)
Supination		60 (45-70)	70 (60-75)	75 (70-85)

*Note.* Data are presented as n/total number of patients or median (interquartile range). MN = median neuropathy; PRWE = Patient-Rated Wrist Evaluation; QuickDASH = Quick Disabilities of the Arm, Shoulder, and Hand; sNCS = sensory nerve conduction study; SWMT = Semmes-Weinstein monofilament test; VAS = visual analogue scale.

**Table 3.** Outcome Measures at the 6-Week, 3-Month, and 12-Month Follow-Ups, Divided by the Presence or Absence of Median Neuropathy (MN) on Orthodromic Sensory Nerve Conduction Studies.

	6 weeks			3 months			12 months		
Variable	MN (n = 30)	No MN (n = 8)	*p*	MN (n = 19)	No MN (n = 18)	*p*	MN (n = 12)	No MN (n = 23)	*P*
SWMT percentage	100 (100-107)	100 (100-105)	0.92	100 (100-107)	100 (100-110)	0.34	100 (95-100)	100 (100-107)	.32
SSD	11/30	1/8	0.39	6/19	4/18	0.71	6/12	2/23	**.05**
QuickDASH	36 (26-56)	33 (23-55)	0.89	18 (9.1-34)	25 (10-40)	0.58	13 (4.5-39)	6.8 (0-16)	.17
PRWE	40 (28-61)	31 (13-65)	0.55	19 (14-35)	23.5(13-47)	0.60	8 (2.3-38)	5.5 (2.5-17)	.55
VAS at rest	0.7 (0-2.6)	1.2 (0-2.0)	0.93	0 (0-0.7)	0 (0-1.1)	0.52	0 (0-0)	0 (0-0)	.73
VAS during activity	3.2 (1.1-5.5)	2.0 (0-4.8)	0.45	1.2(0.7-2.5)	2.0 (0.8-3.7)	0.31	1.0 (0-5.0)	1.0 (0-2.5)	.55
Grip strength percentage	-	-	-	56 (44-72)	58 (41-71)	0.93	78 (68-85)	85 (72-90)	.20
Flexion	48 (40-51)	55 (45-60)		60 (55-65)	55 (45-66)		68 (58-75)	70 (60-75)	
Extension	25 (20-40)	33 (20-58)		45 (35-50)	48 (35-55)		55 (45-60)	55 (45-70)	
Ulnar deviation	20 (15-25)	25 (21-30)		25 (20-30)	25 (20-30)		30 (25-34)	30 (25-30)	
Radial deviation	15 (10-15)	20 (15-20)		15 (15-20)	15 (10-21)		20 (20-24)	20 (15-25)	
Pronation	70 (65-75)	73 (70-83)		75 (70-80)	75 (69-81)		75 (70-80)	75 (70-85)	
Supination	55 (45-70)	63 (53-83)		65 (60-75)	70 (64-76)		70 (70-84)	80 (70-85)	

*Note.* Data are presented as n/total number of patients or median (interquartile range). *P-*value in bold indicates statistical significance. The Mann-Whitney U test was used for comparison of continuous variables. Fisher’s exact test was used for comparison of categorical variables. PRWE = Patient-Rated Wrist Evaluation; QuickDASH = Quick Disabilities of the Arm, Shoulder, and Hand; sNCS = sensory nerve conduction study; SSD = subjective sensory deficit; SWMT = Semmes-Weinstein monofilament test; VAS = visual analogue scale.

There were no significant differences in AO classification or trauma mechanism between the patients with and without MN preoperatively (*P* = .06 and *P* = 1.0, respectively).

Patients with MN at 12 months had median PRWE and QuickDASH scores of 9 (IQR: 4.5-41.5) and 13.6 (IQR: 4.5-43.2), respectively. In contrast, patients without MN at this time point had median scores of 5 (IQR: 1.8-15.2) for PRWE and 3.5 (IQR: 0-12.2) for QuickDASH. These differences were not statistically significant. There were no significant differences in PRWE, QuickDASH, VAS at rest or during activity, grip strength, or SWMT percentage between patients with and without MN at 6 weeks, 3 months, and 12 months postoperatively. Patients with MN had a significantly higher proportion with SSD at 12 months postoperatively compared with those without MN, but at 6 weeks and 3 months postoperatively no significant difference in SSD was observed.

During the follow-up period, plate removal was performed in 2 patients, both of whom had Soong grade 1. Plate removal was scheduled for 3 more patients, 2 with Soong grade 1 and 1 with Soong grade 0.

## Discussion

The main finding of this study was that MN as detected by sNCS was transient in the majority of patients following DRF surgery with a VLP using the VLP. However, 34% of the patients exhibited signs of MN 12 months after surgery.

Nwosu et al^20^ found in a systematic review of randomized controlled trials on VLP for DRFs that median nerve related complications were the most common type of complication after VLP for DRF, with an incidence of 7.9%. Similarly, a retrospective study of 282 patients who underwent surgical treatment with VLP found postoperative hand numbness in 8.5% of the cases (3.2% were considered to have carpal tunnel syndrome).^21^ Only a few studies have investigated MN using NCS after surgery for a DRF using the FCR approach. Demino et al^22^ studied the frequency of MN on NCS following surgical treatment of a DRF using a VLP via the FCR approach. Their cohort included 14 patients with AO type A-C fractures. Their definition of MN was significant latency changes on NCS, indicating a carpal tunnel syndrome. In their cohort, 50% of the patients demonstrated signs of MN on NCS after the injury and before surgery. Six weeks after the surgery, 3 additional patients (21%) demonstrated signs of MN on NCS, but the preoperative changes had resolved in 2 patients (14%). No patients had SSD of the median nerve at the 6-week follow-up. All the patients in our study had AO type C fractures, indicating a more complex fracture pattern and possibly a more extensive fracture, so the results are not directly comparable.

The FCR approach differs from the VCA since it does not expose the median nerve for direct manipulation during the procedure; instead, the nerve is retracted and stretched indirectly. Furthermore, the carpal tunnel is not routinely released during the procedure. The etiology of surgery-related MN is likely different in the FCR approach and VCA. In the FCR approach, the most likely cause of neuropathy is either altered anatomy, swelling, or callus formation leading to increased pressure in the carpal tunnel or traction of the nerve in the trauma or during the surgery. Conversely, when the VCA is used, the carpal tunnel is released and the likely etiology is traction at the initial trauma and/or during surgery.

Gu et al^23^ investigated the results of treating AO type C DRF with combined plating using both volar and radial plates. The volar plate was placed using a median approach, reaching the fracture between the median nerve/finger flexors and the flexor pollicis longus/FCR. Four out of 20 (20%) patients developed transient median neuropathy, which resolved spontaneously within 3 months after surgery. The median approach is similar to the VCA, as the median nerve is exposed and directly retracted during the surgery; and the cohort of Gu et al was also similar to our cohort, since it was composed of patients with AO type C fractures. Gu et al did not perform NCS, but the proportion of patients with SSD was comparable in both cohorts (although in our cohort the SSD did not resolve during the follow-up time of 12 months).

Lattmann et al^13^ compared the FCR approach and the VUA, in which the fracture is exposed ulnar to the finger flexors. The study was retrospective and included 174 patients, and MN was evaluated clinically. At 6 weeks postoperatively, 4.4% of the FCR group and 37% of the VUA group were found to have MN; at 1 year, these proportions had dropped to 0% in the FCR group and 4.8% in the VUA group. The frequency of MN 1-year postoperatively in our study was substantially higher. We speculate that this may be related to all fractures being AO type C fractures, as well as the more direct exposure of the median nerve in the VCA, compared with the VUA where the finger flexors are retracted radially together with the median nerve.

At 12 months, the median PRWE and QuickDASH scores were higher and hand grip strength percentage was lower among patients with MN, indicating a worse outcome compared with patients without MN. These differences were not statistically significant. However, patients with MN showed a significantly higher proportion of SSD at 12 months postoperatively. PRWE and QuickDASH exhibit notable floor effects, meaning that many patients report minimal symptoms or disability.^24^ This likely limits the sensitivity of these measures in detecting the impact of MN. In addition, the limited sample size may have reduced the statistical power to detect significant differences.

In our cohort, the preoperative sNCS showed that 5 out of 35 patients with a valid sNCS had signs of MN and 3 out of 38 patients had SSD. A previous study examining conservatively managed DRF found no electrophysiological signs of axonal loss, but paresthesia in the median nerve territory was experienced by 20% of the patients at 6 to 8 weeks after the fracture and 26% at 12 to 16 weeks after the fracture.^25^ The proportion of patients with SSD was comparable in our study, but the proportion of patients with MN was higher in our cohort. A possible explanation is differences in trauma and fracture severity between the cohorts.

Pain has been identified as the most important outcome measure related to wrist surgery.^26^ The VAS pain scores in our cohort were generally low at 12 months postoperatively, among both patients with and those without MN, and no significant differences were observed. This indicates that the MN does not seem to adversely affect the risk of long-term pain. The wrist ROM in our cohort improved during the follow-up time, and at 12 months postoperatively, it was within the range required for most daily activities.^27^

A key strength of this study is the prospective follow-up of patients, with objective assessment of MN using sNCS, including preoperative testing that allowed identification of trauma-induced MN. However, this study has several limitations. As supramaximal stimulation could not be achieved in the preoperative sNCS, there is a risk that the assessment may have underestimated trauma-related MN. In addition, the lack of a control group restricts the ability to draw definitive conclusions. Comparisons with other studies are also challenging, as differences in cohort characteristics may influence outcomes. If comparisons with existing literature on other surgical approaches had been inconclusive, the addition of a control group using the FCR approach could have been considered in a future study to strengthen the findings.

In conclusion, a notable proportion of patients with DRF treated using the VCA develop median neuropathy. While favorable functional outcomes are generally achievable, we recommend that this approach should be reserved for selected cases where optimal access to the volar ulnar corner is crucial for adequate fracture reduction and stabilization.
